# Antibacterial and Antibiofilm Effect of Low Viscosity Chitosan against* Staphylococcus epidermidis*

**DOI:** 10.1155/2016/9159761

**Published:** 2016-08-18

**Authors:** Inger Sofie Dragland, Håkon Valen Rukke, Ida S. R. Stenhagen, Jessica Lönn-Stensrud, Hilde M. Kopperud

**Affiliations:** ^1^Nordic Institute of Dental Materials (NIOM), Oslo, Norway; ^2^University of Oslo, Oslo, Norway

## Abstract

*Aim*. The aim of this study was to investigate the antibacterial and antibiofilm properties of low viscosity chitosan on* S. epidermidis* growth and biofilm formation.* Methods and Results*. The antibacterial and antibiofilm properties were investigated, during both planktonic growth and biofilm formation. This was performed using different concentrations in media and by coating on polystyrene surfaces. In addition, the bactericidal effect was investigated using a modified direct contact test. The results showed that low viscosity chitosan in media had both a bacteriostatic and bactericidal effect on planktonic growth and biofilm formation of* S. epidermidis* in a concentration dependent manner. Polystyrene discs coated with chitosan reduced both early biofilm formation (6 h) and late biofilm formation (18 h), as confirmed by scanning electron microscopy. The modified direct contact test showed a bactericidal effect.* Conclusion*. This study demonstrated that low viscosity chitosan has a bacteriostatic and bactericidal activity against* S. epidermidis* and that the activity is dependent on the amount of chitosan added. In addition, low viscosity chitosan reduced biofilm formation both when added to media and when coated on polystyrene surfaces.* Significance and Impact of Study*. Low viscosity chitosan could be a contribution to new treatment approaches of biofilm-related infections of* S. epidermidis*.

## 1. Introduction

*Staphylococcus epidermidis*, a Gram-positive, coagulase-negative staphylococcus (CNS) found naturally on the skin and mucous membranes of humans, is considered to be the major cause of infections on indwelling medical devices [1]. CNS, especially* S. epidermidis,* is the most common cause of healthcare-associated bloodstream infections [2]. Frequent use of medical implants has led* S. epidermidis* to develop into an opportunistic pathogen [3]. Biofilm formation is an important virulence factor associated with disease at implant surfaces. “The biofilm way of living” increases resistance against antibiotics and the immune system, making biofilm-related infections difficult to treat [4]. Bacteria in biofilm show up to 1000-fold lower susceptibility to various antimicrobial agents compared to bacteria growing in planktonic culture [5]. Treatment of patients with a chronic infection often involves removal of the infected tissue and replacement of the implant [6]. In addition, isolates of* S. epidermidis* from nosocomial environments are often resistant to multiple antibiotics [7, 8], which highlights the need for finding new modalities to treat and prevent biofilm-related infections.

Several natural compounds prevent biofilm formation with promising results while demonstrating low toxicity to human cells [9, 10]. Chitosan is a natural polysaccharide, composed of glucosamine and *N*-acetyl glucosamine units linked by *β*-1,4-glycosidic bonds. The content of glucosamine is referred to as the degree of deacetylation (DD). Commercial chitosan is produced by partly deacetylating chitin, obtained from the exoskeleton of crustaceans. The molecular weight (MW) of chitosan may range from 50 to over 2000 kDa and the DD from 50 to 95% [11]. Chitosan has chemical, biological, and antimicrobial properties enabling it for applications in a variety of purposes in food production, medicine, agriculture, cosmetics, and biotechnology [12]. Pharmaceutical applications have shown promising results, for instance, using chitosan as a part of artificial skin, in swabs for wound healing and in drug release [13]. Chitosan is suitable for coating and forms films with good strength and barrier properties and has been used in food preservation as a bacterial inhibitor [14, 15].

Chitosan exhibits antimicrobial activity against many different types of bacteria as well as fungi and yeasts [16]. The mechanism behind chitosan's antimicrobial property is linked to the positively charged amino groups. This property is dependent on the degree of deacetylation, which increases the total positive charge of chitosan and the affinity for the negatively charged surfaces of bacteria [17]. In addition, chitosan has shown promising antibiofilm properties, as chitosan-coated dental implants and poly(methyl methacrylate) surfaces can reduce biofilm formation [18, 19]. Upon impregnation of cotton textile with chitosan, the material demonstrated antibacterial effects against isolated staphylococci from normal skin and was suggested as an alternative way to treat skin inflammation [20].

Chitosan is insoluble in most solvents but can be dissolved to a certain extent in dilute acids such as acetic acid, lactic acid, and hydrochloric acid [12]. The poor solubility of chitosan also limits its application. The molecular weight of chitosan has a great impact on the solubility and the viscosity of chitosan in solution. High molecular weight chitosans (HMWC) dissolve more poorly and give solutions of higher viscosity than low molecular chitosans (LMWC) [21, 22]. To overcome this poor solubility, water-soluble chitosan is produced as an oligomer by enzymatic or chemical hydrolysis. Chitosan, LMWC and HMWC, and chitosan oligomers exhibit antibacterial activity against different types of bacteria, but several studies report chitosan to be more effective in inhibiting bacterial growth compared with chitosan oligomers [23, 24]. Also, differences in MW influence the antibacterial effect of chitosan, but this effect seems to depend on the type of bacteria tested and the effect is more influenced by concentration than by MW [20, 23].

The main goal of this study was to investigate the antimicrobial property of low viscosity chitosan (LVC) on* S. epidermidis* growth and biofilm formation, using chitosan dissolved in media and coated on polystyrene surfaces.

## 2. Materials and Methods

### 2.1. *Staphylococcus epidermidis*

A stock culture of* S. epidermidis,* ATCC 35984, was prepared from a −80°C culture in Brain Heart Infusion medium (BHI, Oxoid Ltd., Basingstoke, UK). The culture was incubated aerobically for 6 hours at 37°C before being distributed into tubes and frozen at −20°C. The day before the experiment, the stock culture was diluted (1 : 100 in BHI) and incubated overnight at 37°C. For use in planktonic growth and biofilm experiments, the overnight culture was further diluted (1 : 100 in BHI). For use in the modified direct contact test (DCT), the overnight culture was centrifuged and resuspended in phosphate-buffered saline (PBS) (Lonza, Walkersville, USA) to approximately 1 × 10^8^ CFU mL^−1^.

### 2.2. Test Solutions of LVC

**Figure 1:**
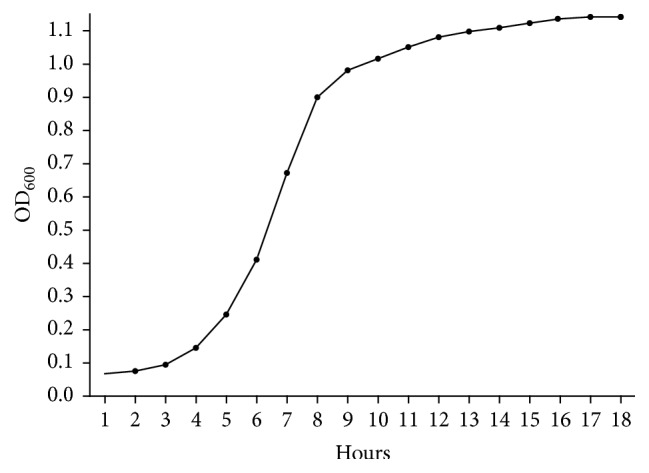
Planktonic growth of* S. epidermidis* in BHI, pH 5.9.

Solutions of LVC from shrimp shells (Sigma-Aldrich 50494, St. Louis, USA, MW 150 kDa, about 80% deacetylated) used for planktonic growth and biofilm formation experiments were made using BHI with pH 5.9. The pH was adjusted with acetic acid (VWR Prolabo, Fontenay-sous-Bois, France) from 7.2 to 5.9 to prevent precipitation of chitosan. To verify good growth at pH 5.9, a growth curve of* S. epidermidis* over 18 hours was made by measuring optical density (OD) at 600 nm in a Multidetection Microplate Reader (Synergy H1, BioTek, USA) (Figure 1).

Solutions of LVC (0–0.02% w/v) in BHI for studies on planktonic growth and growth of biofilm were prepared using a stock solution of 1% w/v LVC in 0.5% acetic acid.

Solutions of LVC (0.25, 0.5, and 1% w/v) in BHI for coating of polystyrene discs and DCT experiments were prepared using 0.5% hydrochloric acid (Merck, Darmstadt, Germany).

### 2.3. Planktonic Growth in Media with and without LVC

The effect of LVC on the planktonic growth of* S. epidermidis* was investigated using different concentrations of LVC in BHI. The control consisted of BHI medium with pH 5.9. The bacteria were incubated aerobically at 37°C for 18 hours. Bacteria suspensions were diluted in PBS and plated onto BHI agar using an automatic spiral plater (Whitley, Don Whitley Scientific Ltd., Shirly, UK) and incubated overnight at 37°C. CFU were counted on the following day using a colony counter (Acolyte, Synbiosis, Cambridge, UK).

The experiments were performed with 4 parallels in 3 separate experiments. Live bacteria were expressed as CFU mL^−1^.

### 2.4. Growth of Biofilm in Media with and without LVC

Biofilm was established on polystyrene discs (d: 13 mm; Thermanox® Plastic Coverslips, Nunc*™*, Rochester, USA) in a 24-well microtiter plate (Sarstedt, Nümbrecht, Germany) in the presence of different concentrations of LVC in BHI. The discs were placed horizontally at the bottom of the wells and grown aerobically at 37°C for 18 hours on a tilt tray (30 tilts/min). At longer growth times, the biofilm tended to detach from the discs. The control consisted of BHI medium with pH 5.9.

The discs with biofilm were washed in PBS to remove the unattached cells and placed in new wells before staining for 10 min with 0.1% safranin (Acros Organics, Glee, Belgium). After staining, the discs were placed in new wells and excess dye was removed by gently rinsing with PBS. The bound safranin was released from the biofilm with 30% acetic acid. Optical density (OD) at 530 nm was measured in a Multidetection Microplate Reader (Synergy H1, BioTek, USA).

The experiments were performed with 4 parallels in 3 separate experiments.

### 2.5. Modified Direct Contact Test (DCT)

The test was performed in a 48-well microtiter plate. The microtiter plate was held in vertical position and the side wall of the wells was coated overnight at 37°C with 50 *µ*L of the LVC solutions. As control, 0.5% HCl was used. The bacterial suspension (10 *µ*L, ca. 10^6^ bacteria) was placed onto the chitosan-coated wells. The plate was left at 37°C for 60 min to allow the bacteria to come into direct contact with the coating. PBS was added to the wells and diluted before 3 × 50 *µ*L drops from each sample were plated on BHI agar and incubated overnight at 37°C. CFU were counted on the following day.

A minimum of 8 parallels from two separate experiments were performed for each of the different concentrations and control. Live bacteria were expressed as CFU mL^−1^.

### 2.6. Growth of Biofilm on Polystyrene Discs Coated with LVC

Polystyrene discs were soaked for 2 hours in solutions of LVC and dried overnight under sterile conditions before biofilm was established. Discs soaked in 0,5% HCl were used as control. In order to adjust the background (blanks), discs coated with LVC solutions corresponding to the test discs were run simultaneously with biofilm experiments.

The discs were washed with PBS before being placed in a 24 well microtiter plate and incubated for 6 and 18 hours on a tilt table (30 tilts/min) for the biofilm of* S. epidermidis* to be established. BHI with pH 5.9 was used in these experiments. After staining with 0,1% safranin and measurements of OD_530_, the results were adjusted with measured OD_530_ from blanks at respective concentrations of LVC.

The experiments were performed with 4 parallels in 3 separate experiments.

### 2.7. Scanning Electron Microscopy

Biofilms and LVC-coated discs were visualized using scanning electron microscopy (SEM). Biofilms and coatings were prepared as described above. After rinsing in PBS, the biofilms and coated discs with bacteria were fixed with 2.5% glutaraldehyde in 0.1 M Sørensens buffer. Samples were dehydrated by rinsing the discs in ethanol, followed by sputter coating with gold palladium. Images were acquired using scanning electron microscopy (Philips XL 30 ESEM, Philips, Eindoven, Netherlands).

### 2.8. Statistical Analysis

The Student *t*-test for parametric independent data and the Mann-Whitney *U* test for nonparametric independent data were used to find significant similarity or difference between the data for a significance level of *p* < 0.05. The analyses were performed with GraphPad Prism version 4.00 for Windows.

## 3. Results

### 3.1. Planktonic Growth in Media with and without LVC

**Figure 2:**
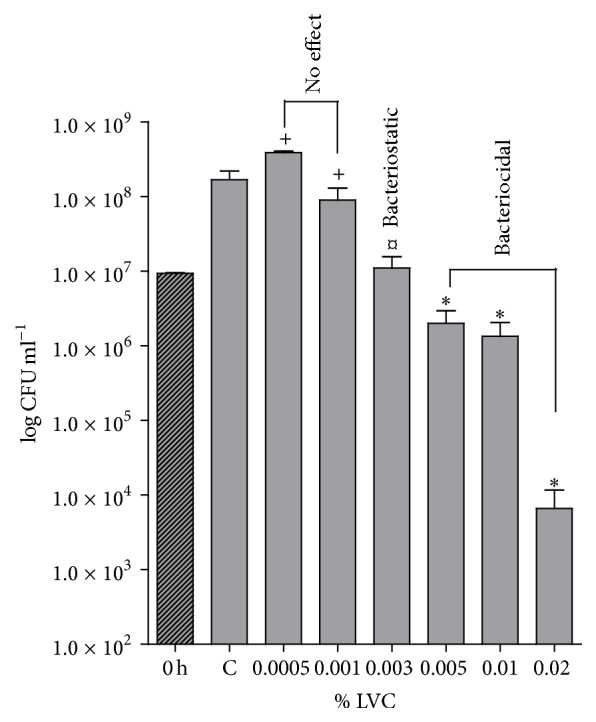
Planktonic growth (CFU mL^−1^) of* S. epidermidis* at 0 hours (before incubation) and after 18 hours of incubation in medium with LVC at concentrations ranging from 0.0005 to 0.02% w/v (*n* = 12). Control (C): medium with pH 5.9 without LVC. *∗*: reduced CFU mL^−1^ compared to C and 0 h; *¤*: reduced CFU mL^−1^ compared to C (but not to 0 hours); +: CFU mL^−1^ corresponding to C.

The bacteriostatic and bactericidal effects of LVC were evaluated using different concentrations of LVC (0 to 0.02% w/v) in medium after 18-hour growth of* S. epidermidis. *Low concentrations of LVC, 0.0005 and 0.001% w/v, did not induce effects on the growth of* S. epidermidis*. However, at higher concentrations of LVC, a bacteriostatic effect on the growth was observed (0.003% w/v), and at concentrations above 0.005% w/v, LVC exhibited a bactericidal effect against* S. epidermidis *(Figure 2).

### 3.2. Growth of Biofilm in Media with and without LVC

**Figure 3:**
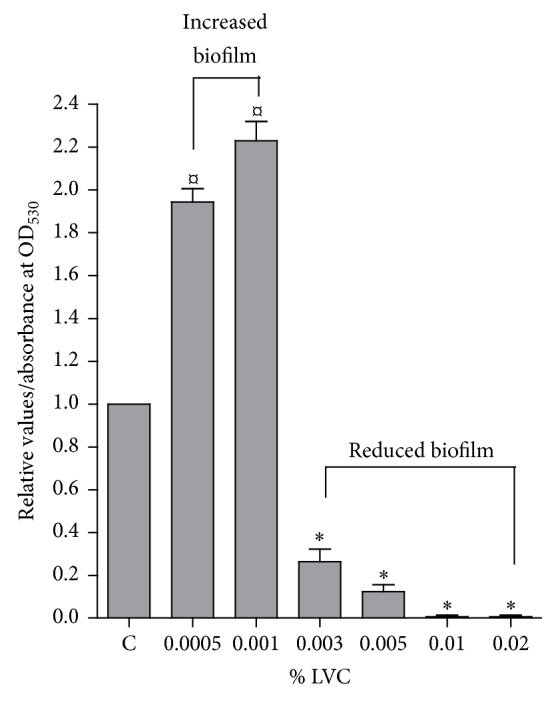
Formation of biofilm of* S. epidermidis* in medium with LVC at concentrations ranging from 0.0005 to 0.02% w/v (*n* = 12). Amount of safranin-stained biomass after 18 hours is expressed as optical density (OD_530_) relative to control (C). Control: medium with pH 5.9 without LVC. *∗*: OD_530_ significantly reduced compared to C; *¤*: OD_530_ significantly increased compared to C.

LVC increased biofilm formation at concentrations below the observed bacteriostatic concentrations from the planktonic experiments. However, increasing LVC concentrations above 0.003% w/v significantly reduced biofilm formation in a concentration dependent manner (Figure 3).

### 3.3. Modified Direct Contact Test

**Figure 4:**
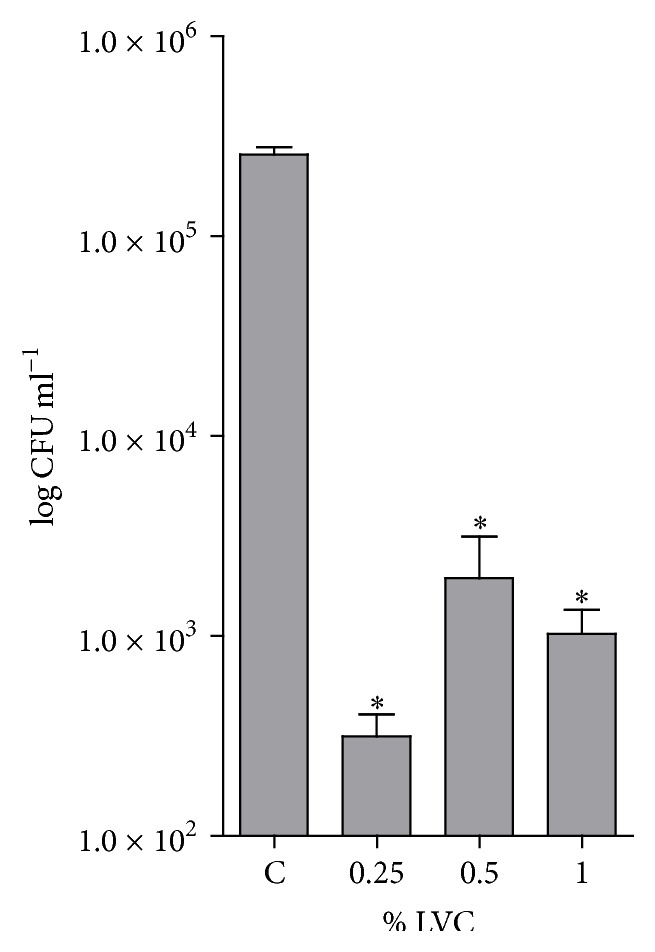
The bactericidal effect of LVC coatings (0.25, 0.5, and 1% w/v) as measured by the modified direct contact test (CFU mL^−1^) relative to control (C) (*n* = 8). Control: 0.5% HCl. *∗*: CFU mL^−1^ significantly reduced compared to C.

The bactericidal effect of LVC-coated surfaces was evaluated using the modified direct contact test. LVC coatings exhibited a bactericidal effect against* S. epidermidis*, with a 2-3-log reduction compared to the control. Increasing the concentration of LVC from 0.25 to 1% w/v when coating the surface did not increase the bactericidal effect of LVC against* S. epidermidis *(Figure 4).

### 3.4. Growth of Biofilm on Polystyrene Discs Coated with LVC

**Figure 5:**
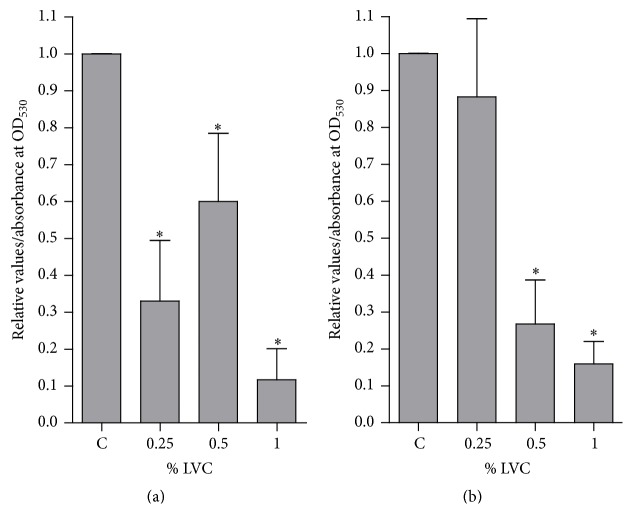
Biofilm of* S. epidermidis* stained with safranin after 6-hour (a) and 18-hour (b) incubation on polystyrene discs coated with LVC (0.25, 0.5, and 1% w/v) (*n* = 12). Amount of biomass is expressed as optical density (OD_530_) relative to control (C). Control: discs coated with 0.5% HCl. *∗*: OD_530_ significantly reduced compared to C.

**Figure 6:**
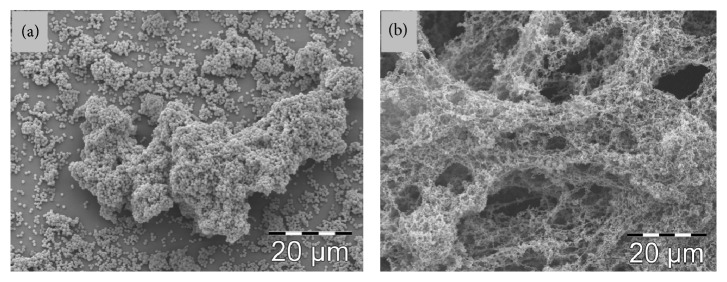
(a) SEM image of polystyrene disc with 6-hour biofilm of* S. epidermidis*. (b) SEM image of polystyrene disc coated with 0.25% w/v LVC with 6-hour biofilm of* S. epidermidis*.

Biofilm formation on coated polystyrene discs using different concentrations of LVC in BHI was investigated after 6 and 18 hours. The amount of biofilm formed after 6 hours at the three concentrations of LVC investigated was significantly lower compared to control. There were no significant differences in the amount of biofilm between the three LVC coatings (Figure 5(a)). After 18 hours, there was significantly less biofilm formation on coatings made with 0.5 and 1% w/v LVC. However, the coating prepared with 0.25% w/v LVC did not reduce biofilm formation after 18 hours (Figure 5(b)). Scanning electron microscopy images of LVC-coated discs showed that LVC was distributed throughout the surface of the discs (Figure 6(b)). Images of biofilm on coated and uncoated discs verified that the LVC coating reduced the amount of biofilm compared to control (Figures 6(a) and 6(b)).

## 4. Discussion

As bacteria on indwelling devises and implants are developing resistance to common antibiotics, new approaches to prevent bacterial growth and biofilm formation are needed. Natural antimicrobial substances, such as chitosan, are therefore relevant alternatives for use as surface coatings to prevent and inhibit biofilm formation of bacteria. CNS, especially* S. epidermidis,* is one of the most notable and frequently found bacteria associated with infected implants [6].

The antimicrobial effect of chitosan has been linked to the positively charged amino groups (NH_3_^+^) of chitosan. These groups are suggested to participate in an electrostatic interaction with the negatively charged groups on the bacterial surface [25]. This interaction may cause damage to the cell wall and alter its permeability and barrier properties [26]. Chitosan's antibacterial and biofilm inhibitory efficiency vary for different species of bacteria [16, 27]. This may partly be explained by differences in the cell wall and expression of different surface molecules, in addition to differences in size, molecular weight, and deacetylation of chitosan itself [28]. In this study, the LVC used had a MW of 150 kDa and chitosan of this size has shown good solubility (9.98 mg/mL) in phosphate buffer and good antibacterial effect at pH 6 [29]. The antibacterial activity of dissolved chitosan is dependent on the pH of the solution, and the antibacterial effect is reduced at higher pH due to less positive charged amino groups [30]. In our study, LVC precipitated out of the media when the pH was raised to 6 and higher. We therefore lowered the pH of the media to 5.9 with an organic acid and recorded good growth of* S. epidermidis*. LVC decreased planktonic growth in a concentration dependent manner when added to the growth medium and exhibited bactericidal effects at higher concentrations. In addition, LVC reduced biofilm formation of* S. epidermidis,* both when exposed to LVC directly in media and when coated on surfaces. The modified direct contact test also confirmed the bactericidal effect of LVC. These results are in accordance with studies on other Gram-positive organisms [20].

Experiments with biofilm and LVC in media showed no antibiofilm effect at low concentrations. Instead, a considerable increase in the biofilm formation was observed. This suggests a stimulating function of LVC at low concentrations, as has been reported for other antimicrobial compounds when applied in low concentrations [31]. Different antibiotics and NaCl have also increased biofilm formation of* S. epidermidis* at potentially toxic concentrations, which was partly explained by the increased expression of polysaccharide intercellular adhesin (PIA), which constitutes the main component of the biofilm matrix [32]. The increased biofilm mass may indicate that these concentrations of LVC may activate a stress response in* S. epidermidis*, which has been shown to increase biofilm formation [33]. In addition, it has been reported that chitosan may lower the metabolic activity of* S. epidermidis* biofilm on intravenous catheters at subinhibitory concentrations [14].

The modified direct contact test used in this study has also been used to evaluate the bactericidal effect of sutures and nanoparticles [34, 35]. We observed that the concentrations tested caused a considerable reduction of the number of CFU, ~99%, compared to control. However, increasing the concentration above 0.25% w/v did not further increase the bactericidal effect of LVC, which may indicate that the surface was saturated. Similarly, results from the coating experiments on polystyrene discs at 6-hour incubation did not depict a concentration dependent behavior for the LVC-coated surfaces at concentrations above 0.25% w/v. Compared with LVC in media, where all bacteria and the whole cell are exposed to LVC, coatings of LVC only come into contact with bacteria in the media through the surface of the polystyrene discs. The SEM images showed that LVC coatings formed a dense network on the surface of the polystyrene discs.

Plexiglas coated with 1% w/v chitosan solution has shown superior antibiofilm effect on* S. epidermidis* compared with several common antiseptic and antibiotic coatings [19]. All three concentrations of LVC coatings on polystyrene discs reduced biofilm formation after 6-hour growth. However, after 18 hours, the lowest concentration of LVC investigated showed increased biofilm compared to early biofilm formation after 6 hours, showing a loss of biofilm inhibitory capacity during longer incubation periods. This phenomenon was however not evident for the higher concentrations of LVC investigated. These results demonstrate the importance of considering the concentrations and the incubation period when investigating the antibiofilm efficiency of chitosan and similar substances.

In this study, we report that LVC has a bacteriostatic and bactericidal activity against* S. epidermidis* and that the activity is dependent on the amount of LVC in media. In addition, LVC reduces biofilm formation both when added to media and when coated on polystyrene surfaces. We propose that LVC can be used as a natural antibacterial material as a coating or by incorporation into different biomaterials.
